# A Comparative Study of Methods for Calculating the Dislocation Density in GaN-on-Si Epitaxial Wafers

**DOI:** 10.3390/mi15080954

**Published:** 2024-07-25

**Authors:** Yujie Yan, Jun Huang, Lei Pan, Biao Meng, Qiangmin Wei, Bing Yang

**Affiliations:** JFS Laboratory, Wuhan 430074, China; yanyujie@jfslab.com.cn (Y.Y.); panlei@jfslab.com.cn (L.P.); mengbiao@jfslab.com.cn (B.M.); weiqiangmin@jfslab.com.cn (Q.W.); yangbing@jfslab.com.cn (B.Y.)

**Keywords:** GaN-on-Si, epitaxial wafers, dislocation density, characterization methods, high-resolution X-ray diffraction

## Abstract

A series of characterization methods involving high-resolution X-ray diffraction (HR-XRD), electron channel contrast imaging (ECCI), cathodoluminescence microscopy (CL), and atomic force microscopy (AFM) were applied to calculate the dislocation density of GaN-on-Si epitaxial wafers, and their performance was analyzed and evaluated. The ECCI technique, owing to its high lateral resolution, reveals dislocation distributions on material surfaces, which can visually characterize the dislocation density. While the CL technique is effective for low-density dislocations, it is difficult to accurately identify the number of dislocation clusters in CL images as the density increases. The AFM technique analyzes surface dislocation characteristics by detecting surface pits caused by dislocations, which are easily affected by sample and probe conditions. A prevalent method for assessing the crystal quality of GaN is the rocking curve of HR-XRD (ω-scan), which calculates the dislocation density based on the FWHM value of the curves. By comparing the above four dislocation characterization methods, the advantages and limitations of each method are clarified, which also verifies the applicability of $D_{B}=\frac{\beta^{2}}{9b^{2}}$ for GaN-on-Si epitaxial wafers. This provides an important reference value for dislocation characterization in GaN-on-Si materials. The accuracy evaluation of dislocation density can truly and reliably reflect crystal quality, which is conducive to further optimization. Furthermore, this study can also be applied to other heterogeneous or homogeneous epitaxial materials.

## 1. Introduction

With the rapid development of modern electronic technology, the performance requirements for semiconductor materials have grown increasingly stringent, yet silicon, the traditional material, which dominates the microelectronics industry, has limited performance in high-frequency and high-power devices. GaN-on-Si materials have attracted the attention of researchers. By integrating GaN with Si, it inherits the stability and applicability of silicon and combines the excellent electronic properties of GaN. Furthermore, the heteroepitaxial growth of GaN on Si substrates is conducive to the realization of large-size, high-quality GaN films [1,2,3,4,5,6,7,8,9,10]. In wireless communications, the high-frequency capabilities of GaN-on-Si make it a key material for next-generation wireless communications technologies (5G and 6G), which are expected to achieve higher data transmission rates and lower signal losses [11,12]. In power electronics, the high power density and miniaturization of GaN-on-Si facilitate efficient and compact device design, which improves power conversion efficiency and reduces energy consumption and cost [4,9,13,14,15].

For heteroepitaxy, the differences in lattice constants and thermal expansion coefficients between the epitaxial layer and substrate lead to the formation of dislocations, which will destroy the crystal structure and lower the performance of the epitaxial layer. Dislocations, as a common defect in semiconductor crystals, mainly affect the electrical and optical properties of semiconductors. In power devices, dislocations can serve as effective channels for carrier leakage, leading to a decrease in breakdown voltage. Meanwhile, dislocations are also important scattering factors, and an increase in dislocation density leads to a decrease in electron mobility. Dislocations acting as non-radiative recombination centers in optical devices will reduce luminescence efficiency. Therefore, it is necessary to efficiently and accurately characterize the dislocation density of semiconductor materials. Dislocation density is a crucial indicator for evaluating the crystal quality of the epitaxial layer, which is of great significance for optimizing preparation processes and promoting material applications [16]. High-resolution X-ray diffraction (HR-XRD) is a non-destructive testing method that is widely utilized for evaluating material crystal quality due to its advantages of simplified sample preparation and convenient analysis. The full width at half maximum (FWHM) of the rocking curve (ω-scan) is typically chosen to calculate the dislocation density of GaN-on-Si epitaxial wafers [17]. Scanning transmission electron microscopy and transmission electron microscopy (STEM-TEM) are commonly used to characterize dislocation distribution and can also be used to evaluate dislocation density when necessary. However, their limitation lies in their small field of view, which can lead to significant statistical errors [18]. Based on various theoretical models, researchers have proposed several formulas for calculating dislocation density [19,20,21,22,23,24,25,26,27,28]. Two of the most commonly used formulas are $D_{B}=\frac{\beta^{2}}{9b^{2}}$, proposed by Kurtz et al. [28], and $D_{B}=\frac{\beta^{2}}{4.35b^{2}}$, proposed by Dunn et al. [26]. Both formulas are grounded in the mosaic model proposed by Gay et al. [27], albeit with differing derivation processes that lead to variations in the parameters. As can be seen from the equation, the dislocation densities calculated by these two formulas can differ by more than a factor of two [25].

In this paper, we provide an in-depth analysis of the dislocation density of GaN-on-Si epitaxial wafers by applying a series of advanced characterization techniques. These techniques include the rocking curve of HR-XRD (ω-scan), cathodoluminescence microscopy (CL) [29,30], electron channel contrast imaging (ECCI) [31,32], and atomic force microscopy (AFM) [33]. Each of these techniques is distinctive and offers comprehensive, accurate, and multi-dimensional information on the dislocation density of GaN-on-Si epitaxial wafers, leveraging distinct physical principles and operational methods. By analyzing the performance test results of GaN-on-Si epitaxial wafers, we can systematically compare the effectiveness of various characterization techniques in detecting dislocation density and gain insight into their technical principles and limitations. This research finding provides a scientific foundation for the analysis of dislocation density in GaN-on-Si epitaxial wafers, further facilitating their application and development in the field of semiconductor devices.

## 2. Experimental Section

In this study, 8-inch GaN-on-Si epitaxial wafers were successfully prepared using the Metal–Organic Chemical Vapor Deposition (MOCVD) technique on the Aixtron AIX G5+ equipment. HR-XRD technology, specifically a Rigaku SmartLab instrument (Rigaku Corporation, Akishima City, Tokyo, Japan), was employed to evaluate the crystal quality of the epitaxial wafers. Additionally, AFM was utilized to characterize the surface morphology, resulting in high-resolution AFM images acquired through the Bruker ICON Dimension system (Bruker Corporation(BRKR), Billerica, Massachusetts, USA). To observe surface dislocation features more intuitively, SEM technology was further adopted, capturing ECCI images in the Backscattered Electron (BSD) mode with the Zeiss Sigma 300 SEM instrument (Carl Zeiss AG, Oberkochen, Germany). Furthermore, a Horiba H-CLU testing instrument (HORIBA Jobin Yvon, Paris, France) was used, combined with CL technology, to comprehensively observe and analyze surface dislocations.

**Figure 1:**
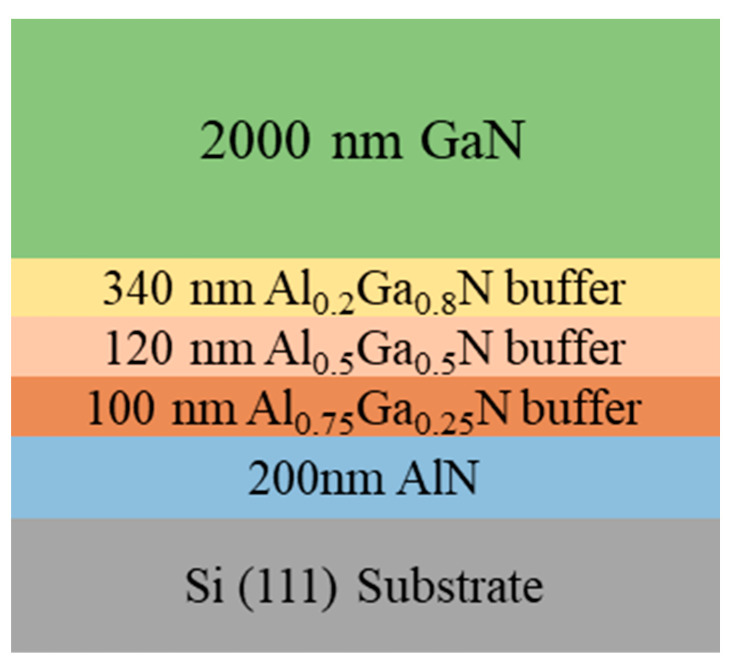
The schematic design of the GaN-on-Si epitaxial growth structure.

Figure 1 depicts the growth structure of GaN-on-Si epitaxial wafers. From bottom to top, a 200 nm-thick high-temperature AlN (HT-AlN) layer as nucleation layer was grown at 1100 °C, and composition gradient Al*_x_*Ga_1−*x*_N layers were used as buffer layer to block dislocations and relieve stress, including a 100 nm-thick Al_0.75_Ga_0.25_N layer, followed by a 120 nm-thick Al_0.5_Ga_0.5_N layer, and finally a 340 nm-thick Al_0.2_Ga_0.8_N layer. The growth temperature of the 2 μm-thick GaN layer was 1050 °C. By changing the growth temperature of the Al*_x_*Ga_1−*x*_N buffer layer, a series of GaN-on-Si epitaxial wafers were obtained. To investigate the influence of dislocation density magnitude on various characterization techniques, three groups of samples with varying dislocation densities were selected and named samples A, B, and C. In order to further enhance the accuracy and reliability of characterization, the chosen detection area was kept consistent when utilizing different techniques for dislocation characterization, and each sample was tested three times and averaged to minimize errors.

## 3. Results and Discussion

**Figure 2:**
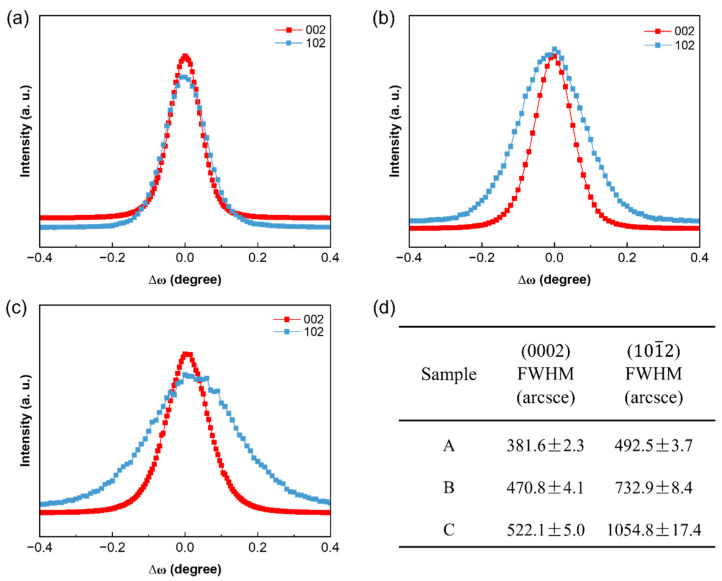
(**a**–**c**) The rocking curves of (0002) and ($10\bar{1}2$) planes of samples A, B, and C, respectively. (**d**) FWHM values for (0002) and ($10\bar{1}2$) planes.

Figure 2 presents the rocking curves of HR-XRD (ω scans) for three groups of samples on the (0002) and $\left(10\bar{1}2\right)$ planes, respectively. Typically, FWHM values can be used to measure dislocation density, with a larger FWHM indicating higher dislocation density. Utilizing Equations (1) and (2), the dislocation densities of GaN-on-Si epitaxial wafers (samples A, B, and C) were calculated with corresponding FWHM values and are summarized in Table 1. For the (0001) plane of GaN films, the Burgers vector **b_1_** = <0001> is defined as the screw dislocation, and the Burgers vector **b_2_** = <11–20> represents the edge dislocation. Consequently, the screw and edge dislocations should be calculated independently, and the total dislocation density is the sum of the two, calculated as follows [22]:

(1) $$D_{dis}=D_{screw}+D_{edge}=\frac{\beta_{\left(0002\right)}^{2}}{4.35b^{2}}+\frac{\beta_{\left(10\bar{1}2\right)}^{2}}{4.35b^{2}}$$

(2) $$D_{dis}=D_{screw}+D_{edge}=\frac{\beta_{\left(0002\right)}^{2}}{9b^{2}}+\frac{\beta_{\left(10\bar{1}2\right)}^{2}}{9b^{2}}$$

It is worth noting that, while the (0001)-plane GaN films also contain mixed dislocations characterized by the Burgers vector **b_3_** = <11–23>, they are not calculated separately, because the mixed dislocation **b_3_** is a combination of **b_1_** and **b_2_**, specifically **b_3_** = **b_1_** + **b_2_**.

**Figure 3:**
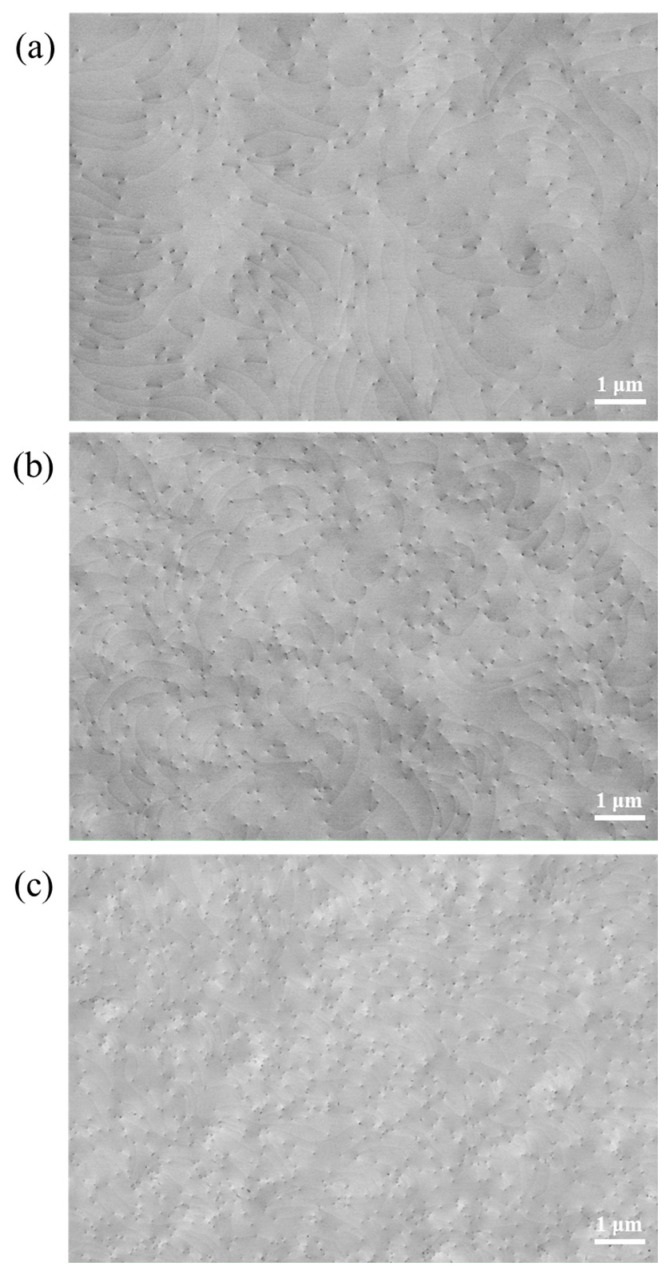
(**a**–**c**) ECCI images of samples A, B, and C, respectively.

Figure 3 shows the ECCI line-scan images of three groups of samples. The ECCI technique, which relies on SEM backscattered electron (BSD) imaging through the electron channeling effect, is suitable for observing and analyzing dislocation structures in materials. When the orientation relationship between the incident electron beam and the crystal grains changes, the BSD signal intensity varies significantly, resulting in a contrast difference in the ECCI image. This contrast difference makes the dislocation regions clearly visible in the image. It is worth mentioning that the signals collected using the ECCI technique primarily originate from a few nanometer ranges on the surface of the material, and its lateral resolution is extremely high, reaching the order of several nanometers. The dislocation points are distinguishable, and the faintly visible lines between the dislocations represent atomic steps on the sample surface. By counting the number of dislocations in the three ECCI images for each group of samples, the dislocation densities of samples A, B, and C were found to be (6.00 ± 0.14) × 10^8^ cm^−2^, (1.06 ± 0.02) × 10^9^ cm^−2^, and (2.12 ± 0.05) × 10^9^ cm^−2^, respectively. The accuracy of ECCI technology in characterizing dislocation density is affected by the surface roughness. For example, when the surface exhibits a rough morphology characterized by step bunching, the identification of dislocations may be limited.

**Figure 4:**
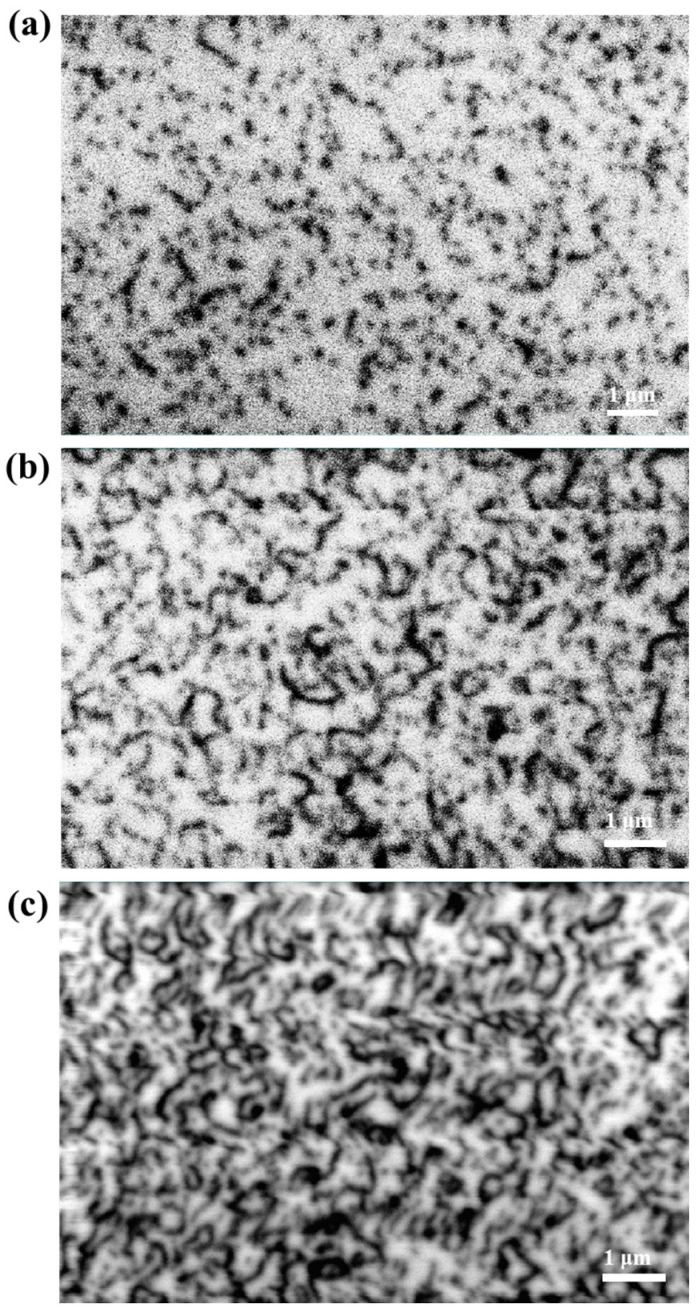
(**a**–**c**) CL images of samples A, B, and C, respectively.

The cathodoluminescence (CL) imaging technique, combining scanning electron microscopy and spectroscopic analysis, exposes the microscopic structure and defects of the crystal by exciting the crystal surface with a high-energy electron beam to produce fluorescence. This technique can sensitively detect the effect of dislocations on fluorescence, allowing the visualization of dislocation characteristics and making it a powerful tool for analyzing the dislocation density of crystals. Figure 4 shows the CL images of three groups of samples taken at an accelerating voltage of 10 kV, where each ‘black dot’ represents a penetrating dislocation. It can be observed that sample A has the fewest and clearest ‘black dots’, indicating the lowest dislocation density. However, a small number of ‘black dots’ overlap and form short, thick black lines due to their proximity, exceeding the resolution of CL, as shown in Figure 3a, and making it difficult to distinguish individual dislocations. The dislocation density calculated from Figure 3a is approximately 7.2 × 10^8^ cm^−2^. With an increase in dislocation density, more ‘black dots’ overlap to form ‘black lines’ in the CL image, leading to significant errors in the counting of dislocation density, as illustrated in Figure 3b (sample B). At a higher dislocation density, the numerous ‘black dots’ representing dislocations overlap, forming tangled black clusters, rendering an accurate count of dislocations impossible. The resolution of dislocation observation using CL is dependent on the diffusion length of GaN minority carriers, as well as the electron beam spot size and accelerating voltage of the CL. Generally, the lateral resolution of CL ranges from tens to hundreds of nanometers, while the longitudinal signal is collected from a depth of hundreds of nanometers. From this perspective, CL resolution is inferior to ECCI and is not suitable for analyzing thin films with high dislocation densities.

**Figure 5:**
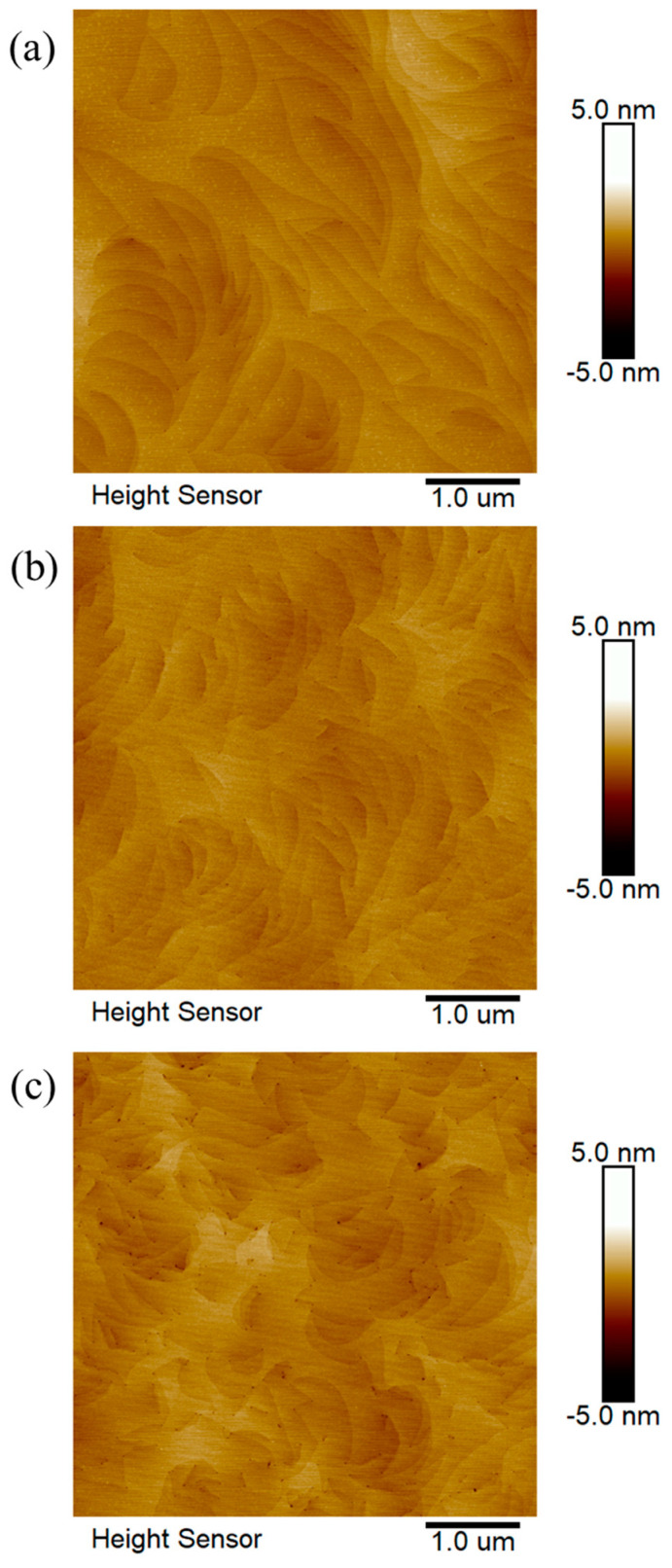
(**a**–**c**) AFM images of samples A, B, and C, respectively.

AFM technology achieves high precision in the characterization of surface topography through the interaction forces between the tip of the probe and atoms on the sample surface. Although the resolution of AFM is influenced by various factors, including the tip radius of the probe and the scanning speed, it typically achieves nanometer-scale resolution, making it a reliable tool for studying the microscopic surface morphology of materials. During the epitaxial growth process, threading dislocations can induce subtle changes in surface morphology, resulting in nanoscale pits. AFM is capable of identifying surface pits induced by dislocations and utilizing them to estimate the dislocation density of the epitaxial layer. However, the scanning range of AFM is relatively small, so collecting images from more different locations is necessary to reduce errors in estimating the dislocation density. Figure 5 shows the AFM images of three groups of samples with a scanning range of 5 μm × 5 μm. The ‘black dots’ in the images are considered to be surface pits caused by the outcroppings of dislocations on the surface. By counting the number of dislocations in three AFM images for each group of samples, the dislocation densities of samples A, B, and C were determined to be (4.40 ± 0.15) × 10^8^ cm^−2^, (7.72 ± 0.17) × 10^8^ cm^−2^, and (1.64 ± 0.34) × 10^9^ cm^−2^, respectively. It is important to note that, because AFM tests the outermost layer of atoms on the sample surface, the AFM may not be able to characterize all surface dislocations due to factors such as the roughness of the sample surface, dislocation outcrops covered with surface-adsorbed atoms, and the tip radius of the probe.

The results of the dislocation densities measured using the CL, ECCI, and AFM detection techniques are compared with those calculated using the FWHMs of rocking curves (ω-scan) according to Equations (1) and (2), respectively, and the results are summarized in Table 2. The ECCI and AFM observations provide dislocation densities at the sample surface, whereas CL shows the dislocation density within a depth range of a few hundred nanometers [30]. Since X-rays can penetrate GaN crystals to a depth of tens of micrometers [23], and the thickness of GaN-on-Si film used in this study is only approximately 2 μm, it can be assumed that the XRD signal originates from the entire GaN epitaxial layer. Since the XRD signal incorporates contributions from the high-defect region located near the AlGaN buffer layer, it may, therefore, lead to an overestimation of dislocation density. As can be observed from Table 2, the dislocation densities calculated based on the FWHMs of rocking curves (ω-scan) are conspicuously higher than those obtained using other detection methods. Moreover, the main factors affecting the FWHMs of rocking curves are the test conditions and the sample itself. For example, the size of the XRD beam spot, dislocation density, film stress state, and wafer warp can cause deviations in the FWHMs. When dislocation density is significant, the broadening of the diffraction peak is primarily attributed to dislocations. Neglecting the impact of other factors in the calculation formula can further exacerbate the overestimation of the dislocation density. As indicated in Table 2, the dislocation densities calculated using Equations (1) and (2) are approximately 1.5 and 3 times higher, respectively, than those counted using ECCI. Despite inherent errors in calculating dislocation density using XRD methods, the estimation of the dislocation density for GaN-on-Si epitaxial wafers, when combined with Equation (2), has relatively smaller errors.

## 4. Conclusions

In this study, GaN-on-Si epitaxial wafers with different dislocation densities ranging from approximately 5 × 10^8^ cm^−2^ to 5 × 10^9^ cm^−2^ were prepared using MOCVD technology, and various characterization techniques were used to conduct detailed comparative studies and an analysis of the dislocation densities in these samples. The ECCI technology, with its excellent high-resolution characteristics, performs well in revealing the dislocations on the material surface, offering an intuitive and efficient method to accurately characterize the dislocation densities of GaN-on-Si epitaxial wafers, particularly those exhibiting step-flow morphologies with low roughness. The CL imaging technique demonstrates remarkable advantages when characterizing samples with low dislocation densities (below 1 × 10^9^ cm^−2^) yet encounters challenges when dealing with samples exhibiting high dislocation densities (above 1 × 10^9^ cm^−2^). The AFM technique, while capable of detecting surface pits caused by dislocations, is often influenced by the surface roughness of the sample and the state of the probe tip, thus limiting its accuracy. In the further XRD ω-scan rocking curve analysis, two formulas based on FWHMs were employed to evaluate the dislocation density. Notably, for GaN-on-Si epitaxial wafers with dislocation densities in the order of 10^8^ to 10^9^ cm^−2^, the calculation results from Equation (2) exhibit a higher degree of consistency with the ECCI, CL, and AFM measurements. This research could offer valuable insights into the dislocation properties of GaN-on-Si epitaxial wafers and their respective evaluation methods.

## Figures and Tables

**Table 1:** The dislocation density of samples A, B, and C with Equations (1) and (2).

Sample	With Equation (1)			With Equation (2)		
	$\frac{\beta_{\left(0002\right)}^{2}}{4.35b^{2}}+\frac{\beta_{\left(10\bar{1}2\right)}^{2}}{4.35b^{2}}$			$\frac{\beta_{\left(0002\right)}^{2}}{9b^{2}}+\frac{\beta_{\left(10\bar{1}2\right)}^{2}}{9b^{2}}$		
	D_screw_ ×10^8^ cm^−2^	D_edge_ ×10^9^ cm^−2^	D_dis_ ×10^9^ cm^−2^	D_screw_ ×10^8^ cm^−2^	D_edge_ ×10^9^ cm^−2^	D_dis_ ×10^9^ cm^−2^
A	2.97 ± 0.04	1.31 ± 0.02	1.61 ± 0.02	1.44 ± 0.02	0.64 ± 0.01	0.78 ± 0.11
B	4.45 ± 0.15	2.85 ± 0.05	3.27 ± 0.07	2.15 ± 0.07	1.34 ± 0.25	1.58 ± 0.32
C	5.62 ± 0.11	6.19 ± 0.20	6.75 ± 0.21	2.72 ± 0.05	2.99 ± 0.97	3.26 ± 0.10

**Table 2:** Comparison of the dislocation densities of samples A, B, and C measured using ECCI, CL, and AFM, and those calculated using the FWHMs of the XRD rocking curves under Equations (1) and (2), respectively.

Sample	ECCI ×10^9^ cm^−2^	CL ×10^9^ cm^−2^	AFM ×10^9^ cm^−2^	XRC Equation (1)	XRC Equation (2)
				$\frac{\beta_{\left(0002\right)}^{2}}{4.35b^{2}}+\frac{\beta_{\left(10\bar{1}2\right)}^{2}}{4.35b^{2}}$	$\frac{\beta_{\left(0002\right)}^{2}}{9b^{2}}+\frac{\beta_{\left(10\bar{1}2\right)}^{2}}{9b^{2}}$
				×10^9^ cm^−2^	×10^9^ cm^−2^
Sample A	0.60 ± 0.01	0.72	0.44 ± 0.02	1.61 ± 0.02	0.78 ± 0.11
Sample B	1.06 ± 0.02	-	0.77 ± 0.02	3.27 ± 0.07	1.58 ± 0.32
Sample C	2.12 ± 0.05	-	1.64 ± 0.34	6.75 ± 0.21	3.26 ± 0.10

## Data Availability

The datasets generated and supporting the findings of this article are obtainable from the corresponding author upon reasonable request.
